# The Role of Leadership Communication in Building Crisis Readiness and Resilient Leadership in Times of Disruption: An Exploratory Study

**DOI:** 10.3390/bs15091260

**Published:** 2025-09-15

**Authors:** Ralph A. Gigliotti, Sonia Alvarez-Robinson

**Affiliations:** 1 Office of Organizational Leadership, Rutgers University, New Brunswick, NJ 08901, USA; 2 Georgia Tech Strategic Consulting, Georgia Institute of Technology, Atlanta, GA 30322, USA; sonia@consulting.gatech.edu

**Keywords:** workplace communication, leadership, crisis, crisis communication, higher education, resilience

## Abstract

In today’s dynamic organizational landscape, workplace communication has become an essential competency for leaders at all levels. With a focus on the narratives used during and after organizational crises—specifically, public examples of workplace communication employed by leaders in a higher education context—this study examines how leaders can cultivate crisis readiness and resilience through strategic communication practices that build trust, convey stability, and strengthen institutional cohesion in times of disruption. Drawing on recent scholarship and public leadership examples, the study introduces a rubric for evaluating resilience narratives that aim to strengthen collective preparedness and adaptability. Framed by the concepts of crisis readiness, resilience, and resilient leadership, this exploratory research highlights how the use of resilience narratives as a form of workplace communication used by leaders can help to bolster collective crisis readiness.

## 1. Introduction

The subject of workplace communication remains a topic of increased scholarly and applied importance for contemporary organizational life. Over the past five years, organizations have gained important insights into the practice of communication. These lessons emerged from navigating a range of complex challenges, including a global pandemic, the rise of remote and hybrid work, the spread of mis- and disinformation, deepening ideological polarization, and the rollback of efforts to promote equity. The convergence of these factors has made effective leadership communication more critical than ever. As defined by Liu et al. (2023), leader communication may be described as “the textual, verbal, and embodied signals that leaders deliver to others, both purposefully and unintentionally, with the power to reveal aspects of leaders themselves, predict leadership outcomes, and affect others” (p. 13). In times of adversity, challenge, and change, members of an organizational community look to leaders to provide vision, inspiration, clear information, and space for community voices to be heard. The messages—intentional and unintentional—shared by leaders take on heightened significance during times of disruption and crisis. This dynamic makes the work of organizational leadership critical and highly complex, and this is certainly the case for leaders in higher education as they respond to the unique pressures impacting the sector and seek to foster greater levels of trust and stability.

The challenges impacting colleges and universities require effective leadership. Furthermore, given the broad array of stakeholders and the diversity of institutions across the sector, the higher education context provides a rich backdrop for understanding the dynamics of leadership communication during periods of crisis and complexity. Furthermore, given the presence of both institutional and environmental challenges, the study and practice of workplace communication remain important for formal and informal leaders. Leaders who possess these capabilities can bolster the readiness and resilience of organizational members. This issue takes on increased significance as we consider the pressing need to create healthy and productive organizations that attract, engage, and retain the talented faculty and staff necessary to deliver exceptional student experiences and ensure success outcomes after graduation.

A focused examination of workplace communication can help to reinforce the linkages to professional civility (Fritz, 2013), positive workplace relationships (Mikkola & Valo, 2020), psychological safety (Edmondson, 1999), and the systems, structures, and processes of contemporary organizations that shape communication practices (Wrench, 2013). Bringing together the expanding research on workplace and organizational communication, along with an analysis of leadership communication narratives presented during and following organizational crises, this article explores the intersection of crisis readiness, resilient leadership, and leadership communication within the context of higher education. More specifically, this research explores the ways in which higher education leaders can help to cultivate a crisis readiness mindset (Jin et al., 2024a, 2024b) through the strategic use of resilience narratives and calls to action, and the implications both resilience and readiness might have for how these organizations navigate crises in an increasingly volatile, complex, and dynamic ecosystem. As Jin et al. (2024a, 2024b) suggest, this focus on readiness as both process and outcome equips organizations and individuals with the agility to respond effectively, adequately, and promptly to various crises.

The findings from this exploratory research—informed by public leadership narratives offered during and in the aftermath of critical incidents in higher education—result in the construction of a rubric for the design and evaluation of these leadership communication narratives. As will be explored in this article, the use of resilience narratives by leaders can help to bolster collective crisis readiness. This form of workplace communication serves an important function for organizations as they navigate disruption in an increasingly volatile, complex, and dynamic ecosystem.

The complexities of contemporary leadership, coupled with the surge of scholarship related to the COVID-19 pandemic, have led to the introduction of many different definitions and perspectives related to readiness, resilience, and resilient leadership. We use the following definitions as a guide for this exploratory study:

Crisis readiness, according to Jin et al. (2024a), “is at least in part a form of efficacy, as it is concerned with motivation and effort for crisis management at the individual-level, crisis team-level and organizational-level, each of which has its unique form of crisis readiness and implies varied degrees of the enactment of crisis management efforts” (p. 7).

Resilience refers to the ability of an individual, organization, or community to adapt, survive and thrive through uncertainty, adversity, and change. Resilience is a state, not a trait, and therefore is developed over time through practices, skills, and strategies to prepare for, respond to, and recover from acute shocks and chronic stressors. As Jin et al. (2024a) suggest, resilience is characterized as a capacity, with some describing it as a dimension of crisis recovery and others highlighting one’s ability to adapt to or withstand during the crisis experience.

The practice of resilient leadership involves the actions of individuals who hold positions of influence, authority, or power within an organization. Those practices include stress response and coping, decision-making, communicating clearly, relating to others, planning and risk management, and adapting to changing circumstances.

This article builds upon the work of scholars who have studied crisis communications, readiness, leadership, and organizational resilience. We offer an additional lens as scholar–practitioners who work in higher education and maintain responsibility for leader development and organizational resilience. We have witnessed first-hand accounts of the challenges that leaders face as they thread the needle amid three intersecting tensions: (1) negotiating both authentic and strategic displays of one’s leadership identity, (2) navigating a complex political landscape that demands an advanced communication acumen, and (3) performing leadership in a way that garners trust, credibility, and confidence. The complexities of contemporary leadership call for leaders to balance these authentic, strategic, and performative identities in their workplace communication—and each of these roles helps to shape the collective resilience and readiness of the communities in which they lead.

We have seen leaders carefully articulate the risks of crisis without creating unnecessary stress, anxiety, and panic among those they lead. We have witnessed leaders who have successfully used communication to reduce and manage risk for both themselves and the organization. At the same time, we have seen leaders struggle, sometimes falter, under the intense pressure of these moments of organizational exigency. We have watched leaders correct, retract, and clarify their initial communication based on reactions from audiences at polar viewpoints. We have also seen leaders step down or be removed from their roles for their failure to effectively thread the crisis communications needle. Our experiences have led us toward a curiosity about the implications for leaders as they dance with these challenges—and the findings from this study highlight some of the ways leaders in higher education may use resilience narratives to help bolster crisis readiness for future crises.

## 2. Research Questions

In alignment with the focus of this Special Issue on workplace communication, the following questions provide a guide for this exploratory study:

RQ1: How do leaders in higher education promote crisis readiness and resilience in their public approaches to workplace communication?

RQ2: What intersections exist among the crisis readiness, resilient leadership, and crisis and risk communication studies that inform leadership communication during and following a crisis?

RQ3: How do leaders’ resilience narratives enhance our understanding of workplace communication during critical incidents?

## 3. Literature Review

### 3.1. Crisis Communication and Crisis Readiness in the Contemporary Workplace

Crisis remains a prominent condition for contemporary workplaces (Roitman, 2013). Crises have the potential to threaten the reputation and mission of organizations and they pose tremendous challenges for those called upon to lead during these critical incidents. Information has the potential to become distorted during times of crisis (Slaughter et al., 2021), and leaders are called upon to respond swiftly, purposefully, and precisely during these periods of acute uncertainty. In their description of “sticky crises,” these moments of disruption “demand not only a near-instant response, but they may require crisis communicators to see possibilities, understand the potential breadth and scope of an emerging crisis, and be ready to change strategy and tactics quickly” (Reber et al., 2021, p. 7). Acts of workplace communication take on heightened significance during these often high-stakes moments in the life of an organization. These moments are often marked by uncertainty and a sense of urgency, and they demand a posture of preparedness that allows for swift decision-making by formal and informal leaders throughout the workplace (Gigliotti, 2022).

One might turn to the crisis communication literature for guidance on research-informed strategies that might be most helpful in responding to these disruptive events or situations (Coombs, 1995, 2023). Indeed, the capacity to identify potential exigencies, harness institutional resources in responding to acute crises, and learn from these critical incidents is central to the work of effective crisis leadership (Gigliotti, 2019). Indeed, the leader’s attitude in a crisis can help to shape how others make sense of and respond to the disruption, while also helping to build trust in the actions and decisions of leaders during these periods of heightened risk and uncertainty (Graffeo & Jin, 2024). The work of risk and crisis communication remains an imperative for leaders at all levels of an organization. Leaders are often expected to offer public statements in response to organizational crises, and these forms of workplace communication, especially when tailored for internal constituencies, serve an important role in shaping perceptions of trust, inclusion, and transparency among key audiences impacted by the crisis at hand (Houlette, 2024; Taylor & Barrera, 2019; Taylor et al., 2023).

Seeger’s (2006) research helps to distill a number of practices associated with effective crisis and risk communication, including the following:

Engage in pre-crisis planning (plan pre-event logistics, coordinate networks, and accept uncertainty).

Model proactive strategies (form partnerships, listen to public concern, and be open and honest).

Demonstrate a strategic response (be accessible to media, communicate compassion, and provide self-efficacy).

Provide clear and consistent communication.

Continuously evaluate and update crisis plans.

These crisis and risk communication practices cut across crisis types, and they certainly take on an elevated significance in dealing with the intersecting challenges of our time. Crises such as the dual pandemics of the COVID-19 health crisis and racial reckoning across the United States, coupled with the outbreak of war in the Middle East, economic uncertainty, and ideological polarization, weigh on organizations of all kinds. Certainly, policy and funding changes initiated by the federal government have had a cascading impact across higher education and have exposed vulnerabilities in communication and engagement with key internal and external audiences. These critical changes have compelled leaders to think boldly and clearly about how readiness might be cultivated within their communities as they prepare for future incidents (Parnell et al., 2010; Parnell & Crandall, 2021). In response to recent crises, the Crisis Communication Think Tank (CCTT) convened a member gathering in April 2023 to explore readiness as a key pillar of crisis management. As Jin et al. (2024a) note, “Readiness refers to the ability of an organization to effectively respond in preparing for and managing a crisis situation. Surpassing mere preparedness, readiness encompasses the initial planning stages to summon a state of action” (p. 2). In their preliminary attempt to define these related concepts, Jin et al. go on to differentiate readiness, preparedness, and resilience using the following emergent themes from their research:

Readiness is a mindset including willingness, awareness, and anticipation.

Readiness is quick and effective implementation of action.

Resources and training are needed to foster readiness.

Preparation is physical (resources, tools, trainings) leading to acquisition of knowledge, experiences, and skills.

Preparation means to have a plan.

Resilience is adapting/withstanding crises.

Resilience is bouncing back/recovering from crisis.

Depicted as both a process and outcome, the ability to navigate uncharted waters demands a readiness mindset, and one that adequately takes account of the individual, the crisis team, and the organization (Jin et al., 2024a). As the authors suggest, “Training and preparedness are not enough to optimally manage a crisis, but with readiness, optimal solutions to crises can be employed” (p. 11).

The exploratory research conducted by Jin et al. (2024a, 2024b) places a focus on the actions needed in responding to crisis, the dimensions of organizational culture that create the appropriate antecedent conditions for readiness, and a consideration of preparedness as a foundation for readiness. This expanding research on this topic positions readiness as a potential new overarching framework that might help to support crisis leadership efforts during episodes of increased scale, scope, and severity (Bundy et al., 2017). Certainly, the complexity of modern organizational life makes the emergence of crises an increasingly pervasive and ubiquitous phenomenon (Perrow, 1984).

The preliminary research on readiness raises a number of central characteristics for leaders to consider. These practices include a commitment to the following:

Prepare relevant plans, resources, and organizational capabilities.

Demonstrate emotional leadership, transformational leadership, and empathy.

Mentally adapt to situations in order to protect an individual from the potential negative effect of stressors.

Maintain awareness of the issues central to public and media debate.

Assess the organization’s commitment to crisis management, including access to needed resources and adequate preparation.

Furthermore, as depicted in the next section, more can be done to explore the relationship between readiness and resilience—and the important role that formal and informal leaders play in helping to bolster both prior to, during, and following moments of organizational crisis through communication.

### 3.2. Resilience

As defined at the outset of this article, the act of resilience refers to the ability of an individual, organization, or community to adapt, survive, and thrive through uncertainty, adversity, and change. Resilience is a state, not a trait, and therefore is developed over time through practices, skills, and strategies to prepare for, respond to, and recover from a myriad of crises. Preparedness and resilience both remain critical in promoting readiness (Jin et al., 2024a, 2024b), and in describing resilience as a capacity, Jin et al. (2024a) go on to note that “Readiness not only exhibits a desire to engage in preparation; it also demonstrates a motivation to develop organizational resilience, mindfully and heuristically” (p. 6).

As supported by the research on communication theory of resilience (CTR), Buzzanell (2010) argues that resilience does not reside in the individual. As she writes, “Rather than an individual phenomenon that someone either possesses or does not, resilience is developed, sustained, and grown through discourse, interaction, and material considerations” (p. 1). Resilience is increasingly characterized and operationalized as an organizational phenomenon (Raetze et al., 2022). Communicative theorizing on resilience focuses on the process of resilience—as a collaborative exchange that is constituted through communicative interactions for which people prepare, continuously learn to enact, and engage strategically (Buzzanell, 2010, 2018). The writing on CTR describes the ways in which individuals, groups, organizations, and communities engage in the active construction and co-construction of resilience (Houston & Buzzanell, 2020). As Wilson et al. (2021) note: “CTR asserts that resilience is both reactive and anticipatory. Although resilience is not conceptualized as a trait, CTR does recognize that some individuals—based on prior experiences and circumstances—may be better positioned than others to enact resilience (or that the same person may be better positioned at some points than others)” (p. 481). According to this theory, resilience is cultivated communicatively and often collectively through the enactment of five sub-processes: crafting a new normalcy; affirming or anchoring important identities during difficult times; using and/or maintaining salient communication networks; looking beyond conventional ways of thinking about and doing life by putting alternative logics to work; and foregrounding productive action while backgrounding unproductive behaviors or negative feelings (Buzzanell, 2010, 2018; Buzzanell & Houston, 2018). Indeed, as explored more fully in a new handbook on the topic, resilience is a “dynamic, communicative process that unfolds across personal, relational, organizational, and societal levels” (Theiss et al., 2025). 

Building out this communication-centered view of resilience further, Barbour et al. (2018) describe how the “organizations and interorganizational systems upon which we depend to keep us safe in a risky world rely on communication” (p. 154). As the authors go on to describe, “Communication choices made in day-to-day work have important implications during emergencies, and the logics underlying those choices may have powerful effects on the forms that reliability, resilience, and safety take” (p. 155). Communication patterns and practices play a critical role in shaping how resilience is nurtured and manifested during crisis response and recovery (Gigliotti, 2025). The communicative enactment of resilience has the potential to play out during each phase of a crisis—in preparing for potential crises that might demand resilience (Doerfel & Prezelj, 2017), in responding to the trigger events that create a sense of loss and disrupt people’s lives (Buzzanell, 2010, 2018), and in cultivating, recognizing, and restoring systems of resilience during the return to stability or normalcy. Drawing upon the existing literature, we view resilience as a collective, organizational, and communicative phenomenon.

One of the ways in which leaders help to build resilience is through the telling and retelling of narratives in their workplace communication. These narratives take on an important meaning-making function during crises that are marked by uncertainty and ambiguity. As Seeger and Sellnow (2016) write,

The lack of clarity and the existence of a communication vacuum and meaning deficit of a crisis create a discursive space that is filled by narratives, often multiple and conflicting. The stories of loss, heroes, victims, hubris, blame, responsibility, recovery, and risk form the basis for a larger structure of sense making and meaning woven around large-scale disasters and crises.

(p. 8)

These narratives and messages invoked in the midst and aftermath of crisis help to reduce uncertainty and encode meaning in events that are often senseless. One of these types of narratives, according to Seeger and Sellnow, is characterized as a renewal narrative. As they write, “The renewal narrative develops after a crisis has created severe disruption to a region, community, or organization. Often basic elements of the establishment have been swept away and components of order and organization, including people, processes, and structures, are gone or no longer function as they have in the past” (p. 81). These narratives have the potential to rally others around a shared sense of hope for the future, and leaders may draw upon these narratives as they call others to action, acknowledge the unifying purpose and values of an organization, or create a compelling and inspiring vision for tomorrow (Seeger & Sellnow, 2016). In many ways, as highlighted in the selected passages to follow, resilience is integrated in many of these renewal narratives. In the midst of disruption, “narratives of renewal describe resilience as both an attribute that allows communities to recover and as a desired outcome from a crisis” (p. 88). As the authors go on to note, “renewal narratives can help to facilitate self-organization and the development of stronger, more effective, and more resilient organizations, communities, and systems” (p. 92).

### 3.3. Resilient Leadership

The resilience of the individual leader can influence the overall resilience of the organization by shaping the responses of those around them. Research has shown that the resilience of a leader has “crossover” effects (Fan et al., 2020) and a “contagion” process (Lin & Liao, 2020) whereby organizational members can adopt the thoughts, beliefs, and actions of their leaders when facing uncertainty, challenges, and change.

Leaders who actively work to develop their own resilience skills, strategies, and practices are more likely to prepare for, respond to, and recover from adversity, crisis, trauma, and other challenges (Southwick et al., 2017). There are strong associations between individual resilience and organizational resilience, tied together through the resilience of leaders (Alvarez-Robinson, 2020, 2021). In their study on the key attributes contributing to institutional effectiveness during and following a crisis, Cameron and Smart (1998) found that most were closely tied to leadership practices. They discovered that “poor leadership appears to be a much more significant factor leading to low institutional effectiveness than the amount of resources available” (p. 81). They concluded that having adequate amounts of people, funding, and tools did not make a difference in the ability to bounce back in the same way that effective leaders did.

Across the literature, five practices were commonly attributed to resilient leadership:

Maintain a focus on the mission and vision of the organization.

Foster connection and cohesion of people.

Model positive emotions (optimism) and a realistic outlook.

Encourage healthy coping mechanisms (flexibility, adaptiveness).

Demonstrate integrity and trustworthiness.

The first practice, maintaining a focus on the mission and vision of the organization, is fundamental to navigating tensions of crisis readiness. Across the literature, the leadership practice that emerged most often as vital to organizational resilience involves a leader’s clear and consistent focus on the mission, vision, and values of the organization (Fan et al., 2020; Southwick et al., 2017; Whitmer & Mellinter, 2016; Moran, 2016). This leadership practice helps organizational members stay focused on their collective purpose and the value they deliver to their constituents. By emphasizing purpose, a leader can help individuals make meaning of a crisis—an essential step in supporting their coping and recovery (Gigliotti, 2024, 2025).

The second practice, fostering connection and cohesion of people, keeps organizational members engaged in the core work of the organization which can help them focus on the good of the organization, rather than the volatility of the world around them (Seville, 2018). During difficult times, leaders should focus on what they can control in terms of resource allocation and support mechanisms (Fan et al., 2020). External threats can increase group cohesiveness and pressures for uniformity (Staw et al., 1981). Leaders can capitalize on the instinct people may have to rally together and bring diverse opinions together while maintaining cohesion of the group toward a common goal (Everly et al., 2013; Southwick et al., 2017).

The fourth resilient leadership practice, encouraging healthy coping mechanisms such as flexibility and adaptiveness, is essential to bolstering crisis readiness and agility when an organization is facing a major challenge or change. In their seminal work, Staw et al. (1981) studied the dysfunctional effects of threat-rigidity response on institutional effectiveness among institutions experiencing organizational decline (Moran, 2016). These early studies revealed a tendency to respond to threats with rigidity, often leading to behaviors such as narrowed focus, oversimplification of information, and a reliance on familiar approaches over creative solutions. Resilient leaders broaden information channels and encourage diverse perspectives to avoid tunnel vision during crises. In a threat-rigidity response, “Communication channels become constricted, only good news is passed upward, and information sharing is attenuated. The emergence of organized, vocal, special-interest groups increases the levels of politicking and conflict among organization members, so employee morale suffers” (Cameron & Smart, 1998, p. 71).

The third resilient leadership practice, modeling positive emotions (optimism) and a realistic outlook, has been shown to be transferable from leader to employees (Fan et al., 2020; Lin & Liao, 2020; Southwick et al., 2017). The words, actions, and emotions of a leader can have a “contagion” effect (Fan et al., 2020) whereby employees adopt the behaviors demonstrated by the leader during the critical incident.

Additionally, as detailed in the fourth resilient leadership practice, encouraging healthy coping mechanisms (flexibility, adaptiveness) goes beyond modeling and involves actively supporting the employees’ development of productive and healthy ways to manage adversity, challenge, and change. Such actions include offering or directing employees to resilience skill-building experiences (Whitmer & Mellinter, 2016; Seville, 2018). In her analysis of communications issued by 38 university presidents in response to the Israel–Palestine conflict, Houlette (2024) found that many leaders faced the challenge of expressing compassion and concern for their communities while avoiding entanglement in public controversy. She noted that the choice to abstain from public comments—or the decision to offer a generic response to the matter—was perceived by others as lacking in compassion. At the same time, some presidents who made statements condemning the violence were perceived as biased or as taking sides in the conflict. This study highlights the challenges leaders face in navigating contentious situations, emphasizing the critical role of workplace communication in their ability to do so effectively.

As detailed in the fifth resilient leadership practice, demonstrating integrity and trustworthiness is critical to establishing mutual trust and respect. Crisis situations often require rapid responses to changing dynamics and quick decision-making by executive leaders. In organizations where deliberation and consensus decisions are core to the culture, expectations must be managed openly. Leadership credibility is fundamental to organizational effectiveness amid crisis and change (Cameron & Smart, 1998; Everly et al., 2013). When organizational members trust their leaders, they have confidence that even if they are not involved in decisions that require quick action, they trust their leaders will make choices that are in the best interest of the organization.

These three intersecting areas of research—risk and crisis communication and readiness, resilience, and resilient leadership—highlight the deep interconnections among them. The complexity of contemporary crises, coupled with the mission-driven focus of affected organizations, underscores the critical importance of effective organizational leadership. Together, these bodies of literature lay the groundwork for a deeper exploration of how leaders may employ resilience narratives as a form of workplace communication to strengthen collective preparedness for future crises.

### 3.4. Theory Dialogue

As the above sections illustrate, leadership communication takes on a heightened significance during times of crisis—moments that are often marked by uncertainty, disinformation, misinformation, and a wide range of emotions among impacted audiences. Communication during times of disruption and in the aftermath of these events can help to shape leadership perceptions and the sense of well-being, safety, and security of those impacted by a crisis. As leaders respond to these critical incidents, their communication directly and indirectly serves an important role in helping members of the community look ahead to where the organization might go next. Therefore, communication during crisis can help to bolster collective resilience in responding to contemporary crises and strengthen the readiness of the community in peering ahead to future crises. For these reasons, the intersecting themes around crisis communication and crisis readiness in the contemporary workplace, resilience, and resilient leadership converge in providing a theoretical base for this exploratory study. By exploring and analyzing these public artifacts of workplace communication, we gain a glimpse into the ways in which leaders demonstrate preparedness and encourage collective resilience both during and following times of organizational disruption. As Liu et al. (2023) suggest,

It is clear that communication is at the heart of leadership. Communication signals a leader’s values, vision, character, and conscience. It is the vehicle through which they transmit who they are and what they want to their followers and other stakeholders. In turn, it influences those stakeholders’ moods, beliefs, motivations, actions, behavior, and performance. In aggregate, this can affect outcomes for organizations, or even countries.

(p. 13)

The study of workplace communication, particularly the communication of leaders during and following acts of crisis, can contribute to our understanding of leadership effectiveness, leader–follower relationships, and the broader organizational outcomes related to readiness and resilience that might result from these visible moments of public leadership.

## 4. Methodology

We engaged in a multi-phased approach to this exploratory study. We first conducted a review of public communication delivered by a sample of higher education leaders. These formal examples of workplace communication consisted of welcome addresses, commencement speeches, and public addresses. All included references to organizational and broader societal challenges. We focused on examples of public communication that directly or indirectly addressed the theme of resilience. In our review, we identified dominant sub-themes related to the resilience process and the ways organizational leaders promoted readiness and resilience. These data were analyzed via an open coding process, beginning first with an identification of resilience-related messages in these public addresses, followed by a constant comparative method (Charmaz, 2006) to investigate relationships among and patterns across the messages.

Building upon emergent themes from our analysis of these resilience narratives and the calls to action issued by executive leaders, we integrated central findings from the literature on crisis readiness, resilient leadership, and crisis and risk communication in the design of a preliminary rubric. We used the rubric to help evaluate the various communications identified in our initial analysis, recognizing that some of the criteria were more easily noticeable in some of the executive leadership communication samples and others would require a more intentional investigation of the critical incidents that preceded or followed these public statements. Importantly, the items depicted in this rubric rely on subjective interpretation of the text, and as the rubric may be applied to other examples of public communication, developing a more robust sampling strategy and engaging in thorough reliability testing of the rubric would be useful. The organization of the content in this preliminary rubric allowed for an initial consideration of each of the following evidence-based practices:

### 4.1. Resilient Leadership (Fan et al., 2020; Lin & Liao, 2020; Southwick et al., 2017)

Maintain a focus on the mission and vision.

Foster connection and cohesion of people.

Model positive emotions (optimism) and a realistic outlook.

Encourage healthy coping mechanisms (flexibility, adaptiveness).

Demonstrate integrity and trustworthiness.

### 4.2. Crisis Readiness (Jin et al., 2024a)

Preparation of relevant plans, resources, and organizational capabilities.

Demonstrate emotional leadership, transformational leadership, and empathy.

Mentally adapt to situations in order to protect an individual from the potential negative effect of stressors.

Maintain awareness of the issues central to public and media debate.

Assess the organization’s commitment to crisis management including access to needed resources and adequate preparation.

### 4.3. Crisis and Risk Communication (Seeger, 2006)

Engage in pre-crisis planning (plan pre-event logistics, coordinate networks, and accept uncertainty).

Model proactive strategies (form partnerships, listen to public concern, and be open and honest).

Demonstrate a strategic response (be accessible to media, communicate compassion, and provide self-efficacy).

Provide clear and consistent communication.

Continuously evaluate and update crisis plans.

For each of the statements included in our sample, one of the researchers applied a rating based on the following criteria:

3—Sample fully meets the best practice
2—Sample partially meets the best practice
1—Sample minimally meets the best practice
0—Sample does not meet the best practice

This phased methodology allowed for an investigation of the selected statements to explore how leaders promote crisis readiness and resilience in these public artifacts of workplace communication. The introductory items included in the rubric also allowed for an analysis of the initial sample speeches in a way that connected back to the central concepts identified in the existing literature. Furthermore, in response to the guiding research questions, the exploratory findings highlight key themes and practices for leaders to consider as they navigate tensions and strengthen crisis readiness in a volatile environment, while also fostering a resilience mindset during times of disruption and urgency.

## 5. Findings

The qualitative analysis of the statements made by executive leaders in our sample led to the emergence of several dominant practices of effective leaders: (a) they tapped into their own internal strength in overcoming obstacles, (b) they faced uncertainty with determination, (c) they demonstrated the courage to question and reimagine, and (d) they shared the struggle in community with others. These four themes, described further in the passages below, draw attention to the collaborative manifestations of resilience among individuals, groups, organizations, and communities (Buzzanell, 2010, 2018; Houston & Buzzanell, 2020; Theiss et al., 2025), and to a view of resilience as both reactive and anticipatory (Wilson et al., 2021). Furthermore, by inviting others to reflect upon and engage in the ongoing work of resilience, these resilience narratives serve an important function in helping to bolster a crisis readiness mindset in preparing for the challenges, setbacks, and disruptions that might lie ahead.

### 5.1. Tapping into Their Own Internal Strength in Overcoming Obstacles

Beginning with this first theme, several leaders used these public remarks to encourage members of the community to tap into their internal strength in responding to future challenges. In their 2024 Commencement address, President of Rice University, Reginald DesRoches, offered the following call to action to graduating students:

Yet, here you are today, more resilient and adaptive than ever and ready to face life with the firsthand knowledge that change and challenges are not meant to be feared but to be hurdled and overcome. So as you stand on the brink of a new chapter in your lives, it’s important to reflect not just on the academic knowledge you’ve gained from your Rice degree but also on the profound lessons you’ve learned beyond the textbooks and outside of the lecture halls. Throughout your time here, you’ve embarked on a journey far greater than simply acquiring knowledge; you’ve learned invaluable lessons about life itself and hopefully about yourself. You’ve encountered a myriad of situations that have tested your resolve, set you back, challenged your perceptions and pushed you beyond your comfort zones. Whether it was tackling a difficult assignment, navigating through personal struggles or grappling with uncertainty, each hurdle has been a lesson in resilience, adaptability and perseverance.

This reminder of resilience was also highlighted by Florida State University President, John Thrasher, in his 2020 State of the University address:

This pandemic has reinforced many of the things I already knew about the people of this university: that we are strong, we are tenacious, and we are resilient. When history looks back on this moment in time, it will see us at our finest… In many ways, the challenges we have faced over the years—the shootings at Strozier Library, three hurricanes, the suspension of Greek Life, and the tragic loss of students to hazing, gun violence and traffic crashes—all have prepared us to respond to the pandemic.

Finally, in one of his bi-weekly messages as interim president of Case Western University, Scott Cowen—someone quite familiar with leading during crisis, having served as president of Tulane University during Hurricane Katrina—drew upon his experience in calling for the resilience of the community:

As I’ve been preparing for my tenure as interim president, I have been reflecting on what it will take for us to emerge better from the pandemic and social unrest across the country. I keep coming back to one word: resilience. The ability to overcome adverse situations, to rebound from hardship, and to adapt in order to move forward are what make us resilient. I have been at the helm of a university under very challenging circumstances before—Hurricane Katrina hit New Orleans seven years into my 16-year tenure as Tulane University’s president—and a key takeaway from that experience is that in order to not just survive but to learn and grow, one has to be resilient. When bad things happen, resilience allows us to not see ourselves as victims but instead focus on what we can control and take charge.

In early July 2025, George Mason University President, Gregory Washington, decided to make a public statement in response to allegations by the federal government that the institution was failing to protect Jewish students from antisemitic actions. He described their actions to “maintain our tradition as a safe and universally welcoming place to study, work, and live,” focusing specifically on issues related to communication, safety, law enforcement, resources and inclusivity. Each of the items described the work they were doing to preserve the integrity of the institution. He did not shy away from the allegations, but rather he defended the institution.

### 5.2. Facing Uncertainty with Determination

The second emergent theme to surface from these public communications included an invitation to face uncertainty with determination, noting the strength and resolve that would serve audiences well in responding to future challenges. In his annual address to the Miami University community, President Gregory Crawford acknowledged the following: “With dedication, perseverance, and confidence, we are empowering ourselves to meet the needs of our time.” Writing about the impact of the pandemic on the institution, he noted that “we will undergo—not only a recovery—but a renaissance that advances our mission and elevates our impact … We will not retreat or retrench.”

We see similar themes in the remarks by former President of California State University, Sacramento, Robert Nelson, in his 2021 Fall address. With a nod to Bruce Springsteen’s “The Rising,” Nelson invoked the following lyrics in his introductory remarks: “Can’t see nothing in front of me. Can’t see nothing coming up behind. Make my way through this darkness. I can’t feel nothing but this chain that binds me. Lost track of how far I’ve gone. How far I’ve gone, how high I’ve climbed.” Referring to the “Hornet heroes” who helped the institution in responding to the pandemic, Nelson goes on to recognize the individual groups who played a key role in the response. He goes on to suggest: “Yes, football is back. So are dance and theatre. And let’s not forget concerts and music. ‘Come on up for the rising.’” By invoking “the rising,” another reference to Springsteen, Nelson points to the resilience of the community that would help it to face the lingering uncertainty posed by the pandemic in the period to follow.

In May 2025, UVA President Jim Ryan spoke to the alumni at Reunions weekend about the challenges that UVA and higher education were facing. He courageously described the funding cuts, policies related to international students, and prohibitions on DEI programs. He said, “The waters are undoubtedly choppy at the moment for higher education, including for UVA. But UVA is a very strong ship.” He went on to provide an inspiring picture of a resilient organization, ready to withstand the forces of change in front of it. He celebrated the achievements of the institution and provided an honest but encouraging outlook: “We are also not completely insulated by the world outside of Charlottesville. And there may be difficult choices and decisions to make ahead. But make no mistake, this is a remarkable institution and one worth protecting.” Then just two months later, President Ryan announced his resignation after the Department of Justice alleged that UVA had not fully dismantled their DEI programs. In his farewell statement, Ryan acknowledged the following: “We have faced some extraordinary challenges over the last seven years. We made it through and were able to thrive because we provided strength to and drew strength from each other. If you continue to support and rely on each other, there is no obstacle too large for this community to overcome and no goal too ambitious to reach.” He did not mention explicitly the reasons for his departure, only his confidence in the community to navigate these challenging times together.

### 5.3. Demonstrating the Courage to Question and Reimagine

The third theme speaks to the ways in which individuals and groups may feel compelled to question and reimagine in their response to the fear, destruction, and uncertainty posed by crises. This act of resilience could help to position the organization and its members in a way that allows for the forging of a new path.

In her inauguration address, former president of Harvard University, Claudine Gay, suggests the following:

Not four hundred yards from where I stand, some four centuries ago, four enslaved people—Titus, Venus, Bilhah, and Juba—lived and worked in Wadsworth House as the personal property of the president of Harvard University. My story is not their story. I am a daughter of Haitian immigrants to this country. But our stories—and the stories of the many trailblazers between us—are linked by this institution’s long history of exclusion and the long journey of resistance and resilience to overcome it. And because of the collective courage of all those who walked that impossible distance, across centuries, and dared to create a different future, I stand before you on this stage—in this distinguished company and magnificent theatre, at this moment of challenge in our nation and in the world, with the weight and honor of being a ‘first’—able to say, ‘I am Claudine Gay, the president of Harvard University.’ Their courage, that courage, is what I want to reflect on today: The courage of this University—our resolve, against all odds—to question the world as it is and imagine and make a better one. It is what Harvard was made to do.

Gay went on to pose the following questions to the Harvard community: “What we offer to the world will depend on Harvard’s courage—our courage—to ask two questions that propel our work—Why? and Why not? And it will depend on the courage to answer, with confidence, two others: Why here? and Why now?” Just six short months later, Dr. Gay resigned in response to intense criticism around what she described as “personal attacks and threats fueled by racial animus.”

Two years after Gay’s departure, her successor faced new challenges in a public standoff with the new federal administration. In an April 2025 letter to the Harvard community, President Alan Garber underscored the value of Harvard’s research, condemned government overreach, and described the commitments he was making to their community.

“As we defend Harvard, we will continue to: nurture a thriving culture of open inquiry on our campus; develop the tools, skills, and practices needed to engage constructively with one another; and broaden the intellectual and viewpoint diversity within our community; affirm the rights and responsibilities we share; respect free speech and dissent while also ensuring that protest occurs in a time, place, and manner that does not interfere with teaching, learning, and research; and enhance the consistency and fairness of disciplinary processes; and work together to find ways, consistent with law, to foster and support a vibrant community that exemplifies, respects, and embraces difference.”

Garber’s communication here displays strength, determination, and courage to question and challenge powers he sees as a threat to their mission.

### 5.4. Sharing the Struggle in Community with Others

Finally, the fourth theme of significance related to these public messages of resilience involves the recognition of the power and importance of coming together as a community. Organizations rely on the interdependence of their networks, and their people to stand in support of one another and acknowledge the shared contributions to building resilient organizations in the face of adversity.

In his 2021 opening exercises address, Princeton University President Christopher Eisgruber reminds his audience of the following lesson on resilience in responding to the “hidden challenges in our lives.” As he suggests, “When you are dealing with your own challenges it can be helpful to remember that you are not alone. Conversely, as you interact with people around you I hope you will keep in mind that they may be dealing with troubles that you cannot see or that they are not ready or able to share. That condition is part of what makes us human, and one of many reasons why we need to treat each other humanely.” Describing the importance of humility, Eisgruber reminds his audience of the following: “We are all, all of us fragile and flawed, yet we can reach for the stars and do tremendous good. That astonishing combination of weakness and courage is part of what defines the human condition.”

Again, the written remarks by Cowen highlight the shared work of collective resilience: “I have seen resilience everywhere around me during my first few weeks as interim president—whether it was at Homecoming or my meetings with the various schools and student organizations—and when I think about the combined resilience within our community, anything seems possible.”

In April 2025, 250 university presidents joined in developing A Call for Constructive Dialogue in response to actions being taken by the federal government against higher education institutions. In this statement, they demonstrated resilient leadership by focusing on the mission of higher education, and passionately expressed the urgency of the moment in a call for productive discourse, all the while modeling courageous conviction to speak their truth.

American institutions of higher learning are essential to American prosperity and serve as productive partners with government in promoting the common good. Colleges and universities are engines of opportunity and mobility, anchor institutions that contribute to economic and cultural vitality regionally and in our local communities. They foster creativity and innovation, provide human resources to meet the fast-changing demands of our dynamic workforce, and are themselves major employers. They nurture the scholarly pursuits that ensure America’s leadership in research, and many provide healthcare and other essential services. Most fundamentally, America’s colleges and universities prepare an educated citizenry to sustain our democracy.

(*A Call for Constructive Engagement*, 2025)

These four emergent themes—(a) tapping into one’s internal strength in overcoming obstacles, (b) facing uncertainty with determination, (c) demonstrating the courage to question and reimagine, and (d) sharing the struggle in community with others—provide a glimpse into the different ways that executive leaders describe the work of resilience. These limited examples of formal workplace communication are tailored to unique internal and external audiences—serving an important role in helping others make sense of disruption and bolstering collective readiness in response to future critical incidents.

## 6. Discussion

The initial coding of resilience-related narratives and public addresses highlighted the various ways in which leaders call attention to the cultivation of a resilience mindset in the midst and aftermath of collective disruption. Additionally, these messages seem to play an important role in helping their communities stand ready for whatever crises might lie ahead. Insights from this initial phase of research informed the development of a preliminary rubric for evaluating leadership messages, enabling us to analyze their connections to readiness, resilient leadership, and risk and crisis communication.

The development and pilot testing of our rubric provided a structured framework for examining examples of readiness and resilience through the lens of evidence-based practices. For this article, we applied the rubric to a sample of leadership communications that exemplified resilience, scoring each narrative based on the themes identified in our literature review. As anticipated, the results revealed a positive relationship between leadership resilience and effective crisis communication. Moreover, the rubric proved useful in highlighting the interconnectedness of readiness, resilient leadership, and risk and crisis communication.

It is our hope that others may use the rubric in evaluating other public forms of workplace communication delivered during and following crisis events and situations. The rubric may also serve as a guide for leaders in building their crisis readiness and response capabilities and plans. By embedding resilience-oriented themes into the content, tone, and delivery of leadership communications, leaders can facilitate the transfer of resilience to members across the organization (Fan et al., 2020).

Given the increasingly volatile nature of our world and the overwhelming sense of fatigue, burnout, and disengagement experienced by people in organizations, the cultivation of resilience and readiness remains a leadership imperative (McClure, 2025). Messages of resilience and renewal in the face and aftermath of a crisis can contribute to the cultivation of a collective readiness mindset, leading to increased potential for resilience when faced with future disruptive events. As Jin et al. (2024a) suggest, “a state of readiness empowers crisis managers to confront unforeseen challenges and obstacles” (p. 2).

Leaders play an important role in fostering optimism and a positive outlook by reminding community members of their strengths and capabilities to endure challenges. These messages of resilience invite others to stand ready for whatever crises might lie ahead. As the various examples highlighted in this article suggest, these stories, narratives, and insights related to resilience remind us of (a) our inner strength and experience in overcoming past obstacles, (b) our ability to face a volatile and uncertain world with determination and conviction, (c) our courage to question, reimagine, and chart a new path forward, and (d) our sense of connection to others as we tap into the collective spirit of the communities of which we are a part.

## 7. Implications

Despite the focus on higher education examples at the presidential level in this exploratory study, we suspect that many of the implications could be relevant for leadership communication across sectors and levels. While resilience is most often associated with bouncing back from adversity, and the ability to return to a steady state, we have seen that resilience is central to the process of readiness in addition to being an outcome. As readiness has been constructed as “the mental state of being willing to engage the crisis” (Jin et al., 2024a, p. 1), we recognize that resilience and readiness can be symbiotic, whereby resilience can be both a process and outcome of readiness, and readiness is a process and outcome of resilience. Both are mutually reinforcing in crisis preparation, response, and recovery within and beyond higher education—and the writing on workplace communication helps to support inquiries exploring these themes of interest.

Modeling resilient leadership through effective crisis communication is an art that requires intention and practice. Leaders must authentically and proactively communicate about risks and threats in the face of crisis without catastrophizing or stoking panic. They must leverage the strengths of organizational cultures that value shared governance, thoughtful deliberation, and the collaborative exchange of ideas while also responding quickly and nimbly. The research shows that maintaining a focus on mission and vision, fostering connection and cohesion, modeling positive emotions and a realistic outlook, encouraging healthy coping, and demonstrating integrity and trustworthiness are critical to building broader organizational capabilities. In response to the exploratory themes shared in this paper, colleges and universities, along with other organizations, should continue to implement resilience-building communication strategies into institutional and association crisis management training programs.

To foster readiness, leaders should engage their teams and organizations in preparing plans, resources, and organizational capabilities, while also demonstrating emotional intelligence and empathy in their formal and informal acts of workplace communication. Leaders have a unique role in helping members of their community to mentally adapt to changing dynamics as they provide access to needed resources. They must maintain awareness of the issues central to public and media debate while taking care not to put themselves at the center of conflict. This can be a difficult needle to thread, particularly when the crisis is born from struggles for power and dominance. Some mission-focused organizations today insist on institutional impartiality or institutional restraint as a strategy to remain viable and out of the spotlight of public controversy. The American Red Cross’ Statement of Impartiality says, in part, “Just as disasters don’t discriminate in terms of whose lives they destroy; the Red Cross doesn’t discriminate in whose lives we help rebuild. The Red Cross is a safe and secure place for everyone in need after a disaster” (American Red Cross, n.d.).

Impartiality, neutrality, and objectivity can be distinguished from institutional restraint, whereby the leaders of an organization choose to hold back communication for fear of being caught in a no-win situation. President of Princeton University, Christopher Eisgruber, described their tradition of institutional restraint in a memo posted on his webpage in November 2022. He said, “my principal responsibility as president is to ensure that the University remains an impartial forum for vigorous, high-quality discussion, debate, scholarship, and teaching. I therefore have a presumption against commenting on social, moral, or political topics” (Eisgruber, 2022). This approach to institutional restraint appears to be a growing trend across colleges and universities, especially in navigating a shifting political terrain. By leaning upon one’s mission and values, leaders of these organizations may find this strategy to be useful in amplifying one’s core purpose and avoiding controversies in dealing with some of the polarized issues of our time.

## 8. Limitations and Future Research

In their 2022 symposium, Shek and Wilkinson (2022) encouraged researchers to use instruments to assess leadership behaviors and to consider how these behaviors impact employee resilience. Furthermore, as the authors note, “researchers need to develop conceptual models on the consequences of resilient leadership on leaders, followers, and organizations, and conduct empirical studies to examine such theoretical postulations” (p. 52). The items depicted in the rubric are an initial attempt to link key themes in the existing literature with the analysis of these public examples of leadership communication. In using the rubric to conduct a preliminary evaluation of the public speeches, the researchers found the concepts to be relevant and useful for analysis. However, future use of the rubric should consider usage of the rubric with other instruments designed to assess broader leadership behaviors, along with more thorough reliability testing and consistency checks to enhance the rigor and credibility of this conceptual tool.

This study focuses specifically on the analysis of a limited sample of prepared public statements between 2020 and 2025. These selected texts are primarily ceremonial or public addresses, which are often highly scripted and lack the immediacy and complexity of real-time crisis communication. This study does not take into account the more extemporaneous examples of crisis communication used by leaders nor is it exhaustive of examples representing the diversity of institutional types. In the narratives and calls to action analyzed for this study, we took note of aspects of resilient leadership that may directly or indirectly contribute to crisis readiness. Nearly all initial public communications selected for analysis were speeches prepared for celebratory and milestone events such as commencement, annual addresses, and convocation. The tone of these events is intended to uplift and inspire, so it stands to reason that messaging of overcoming adversity is such a prominent theme. These examples are not fully representative of the types of crisis communication that require leaders to quickly issue messaging in preparation for, or response to, a major crisis.

The central findings described in this paper are derived from an exploratory analysis of communication conducted in the middle and aftermath of crisis. Future research can explore the extent to which these themes may apply to other phases of crisis communication (such as pre-crisis). Additionally, by conducting interviews with leaders as part of an expanded study, it may be of interest to explore the relationship between these emergent themes and the communicative intent of the leaders, such as reassurance, explanation, clarification, motivation, mobilization, repair, or healing.

In future studies we aim to use our rubric to examine more recent communications associated with major adversities, challenges, or change, and to explore further the ways in which executive leadership communications help to bolster readiness and resilience during these times of disruption and, at times, despair. A more robust testing of the inter-rater reliability of this conceptual tool will help to refine the scoring criteria. Additionally, the selected methodology for this study focuses on public communications artifacts and not on perceptions of impact among recipients of these messages. This expanded approach to future research can help in evaluating whether these leadership narratives were perceived as meaningful or effective by organizational members, and may go so far as to explore the relationship between the invocation of the themes described in this paper and potential outcomes of interest, such as organizational outcomes, perceptions of employee feedback, or media response to the crisis. Importantly, future scholars may build upon these findings to consider some of these missing variables in future research and may pursue alternative methodologies such as interviews or focus groups to better understand the perceptions and lived experiences of individuals impacted by these public messages of resilience.

## 9. Conclusions

As noted in the call for submissions, the guest editors highlight Mikkola and Valo’s (2020) definition of workplace communication as encompassing interpersonal communication, interpersonal relationships, and social interaction as the central features of workplace communication, situating workplace communication “at the intersection of interpersonal and organizational communication” (p. xi). In attempting to establish the intellectual boundaries around workplace communication, we would advocate for the inclusion of leadership resilience and crisis communication as two areas ripe for additional research through the lens of workplace communication. As described throughout this article, organizations and their leaders must navigate several tensions as they help followers make sense of crisis and stand ready to respond to the pressures of these moments. The preliminary writing on readiness and resilience introduces the inherent connections between these two concepts, and this exploratory examination of the speeches and narratives used in various public communications contributes to the conversation on this important subject. In short, as supported by the findings of this study, it is through the direct and indirect invocation and cultivation of resilience that leaders might help to encourage a readiness mindset. The looming threat of future crises elevates the importance of this issue for leaders at all levels—and raises the stakes for future workplace communication scholarship on this topic.

## Data Availability

The original contributions presented in this study are included in the article. Further inquiries can be directed to the corresponding author.
